# Prevalence and incidence of cognitive impairment in an elder Portuguese population (65–85 years old)

**DOI:** 10.1186/s12877-020-01863-7

**Published:** 2020-11-16

**Authors:** Ricardo Pais, Luís Ruano, Carla Moreira, Ofélia P. Carvalho, Henrique Barros

**Affiliations:** 1 EPIUnit - Instituto de Saúde Pública, Universidade do Porto, Rua das Taipas, n° 135, 4050-600 Porto, Portugal; 2 Departamento de Epidemiologia Clínica, Medicina Preditiva e Saúde Pública, Faculdade de Medicina da Universidade do Porto, Alameda Prof. Hernâni Monteiro, 4200-319 Porto, Portugal; 3 Unidade de Saúde Familiar Lusitana, Av. António José Almeida, 3514-511 Viseu, Portugal; 4 Departamento de Neurologia, Hospital de São Sebastião, Centro Hospitalar de Entre o Douro e Vouga, Rua Cândido Pinho, 4520-211 Santa Maria da Feira, Portugal

**Keywords:** Cognitive impairment, Prevalence, Incidence, Population-based cohort, EPIPorto

## Abstract

**Background:**

The increase in average life expectancy increases the risk of illness and frailty in the elderly, especially in the cognitive arena. This study has the objective to estimate the prevalence and incidence of cognitive impairment, in a representative sample of 65 to 85 years old followed for a mean period of 6-years.

**Methods:**

Subjects aged 65–85 years (*n* = 586) were screened at baseline (1999–2004) to estimate the prevalence of cognitive impairment using the Mini-Mental State Examination. A total of 287 individuals with a normal MMSE at baseline were reassessed after 6.2 mean years (± 4.30 years) to evaluate the incidence of cognitive impairment, defined as scoring below the age and education-adjusted MMSE cut-off points adapted for the Portuguese population. We did not exclude Dementia.

**Results:**

The baseline prevalence of cognitive impairment was 15.5% (95% CI: 12.7–18.7). Higher in women (18.9%; 95% CI: 14.9–23.3), that in men (10.4%; 95% CI: 6.7–15.1). Increased with age and was highest for participants without any schooling. The overall incidence rate was 26.97 per 1000 person-years; higher in women (33.8 per 1000 person-years) than in men (18.0 per 1000 person-years). Higher for the oldest participants and those with no schooling. Taking the standard European population, we estimated a prevalence of 16.5% and an incidence of 34.4 per 1000 person-years.

**Conclusion:**

The prevalence of cognitive impairment in Portugal is within the estimated interval for the European population, and the incidence is lower than for the majority of the European countries. Women, senior and elders without education have a higher risk of cognitive impairment. In our sample, neither employment nor marital status has a significant effect on cognitive impairment.

**Supplementary Information:**

The online version contains supplementary material available at 10.1186/s12877-020-01863-7.

## Background

The ageing of the world population is a demographic trend that will intensify in the coming decades. Eurostat projects that by 2050 Portugal will be the European country with the highest percentage of people aged 55 years or more (47%) [1]. The growing number of older people poses health challenges such as increasing the prevalence of disease and disability in the elders, especially the burden of cognitive dysfunctions [2]. Cognitive impairment increases the risk of dementia and mortality in the elders [3, 4]. It is characterised by individuals having more difficulties with memory, learning, and the ability to focus on a task, than would be normally expected for the individual’s age and educational level [5]. It ranges from mild deficits that are not clinically detectable to dementia [5]. It has a social impact and is associated with other pathologies, such as Alzheimer or Dementia [6, 7]. Age, sex and level of education are considered risk factors for cognitive impairment [4]. Continued professional activities may be protective against cognitive decline [8] however there is a lack of information about the impact on cognitive function of postponement of retirement age. Also, changing demographics characteristics added to a higher divorce rate increases the number of older people living alone, especially women, which traditionally already presented with an increased risk of cognitive decline [1, 9, 10].

Reports on the prevalence and incidence of cognitive impairment, as well as its relation with comorbidities and sociodemographic factors, are essential for public health and clinical care policy. They are necessary to allow primary and secondary prevention measures within the healthcare system.

In Europe, published studies report the prevalence of cognitive impairment to be between 5.1 and 24.5% [11–16], whereas in North America, the estimated cognitive impairment prevalence ranges from 13.8 to 28.3% [17–19]. In Europe reports that used the Mini-Mental State Exam for cognitive impairment evaluation in samples with the same age characteristics as ours estimated cognitive impairment prevalence between 7.7 and 33.1% [12, 16, 20]. The incidence of cognitive impairment ranges from 56.5 to 76.8 per 1000 person-years in Europe [16, 20, 21] and from 41.8 to 65.4 per 1000 person-years, in North America [22–24] In Portugal, previously published studies report a prevalence of cognitive impairment ranging from 9.3 to 12.0% [10, 25, 26] and as far as we know, the incidence is unknown.

## Methods

### Aim

This study aims to estimate the prevalence and incidence of cognitive impairment after 6.2 mean year’s follow-up assessed using the Mini-Mental State Exam (MMSE) in a cohort of city dwellers from Porto, Portugal, aged 65 to 85 years old, and to evaluate the impact of age, sex, schooling, retirement and civil status in cognitive function. For the main variables of interest, we hypothesize that cognitive impairment prevalence and incidence are similar to other European countries.

### Study population

**Fig. 1:**
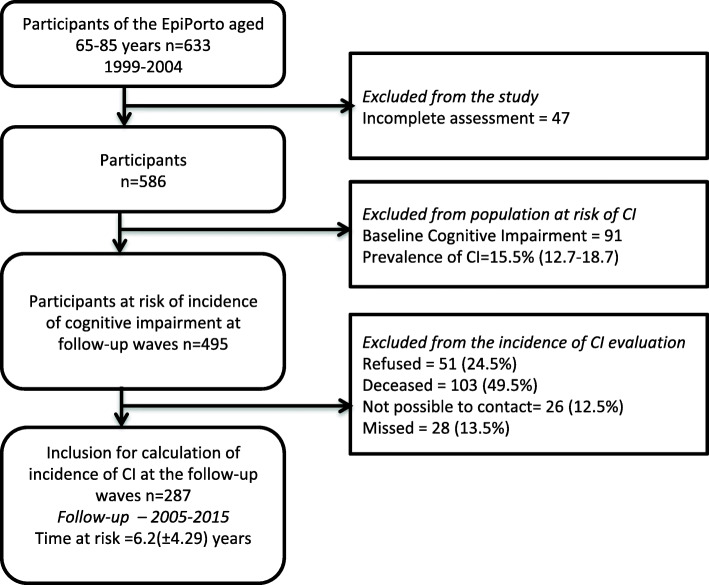
Flow-chart of participants through the steps of the study and final results on the frequency of cognitive impairment

The EPIPorto cohort study design and methodology have been published previously [27, 28]. In brief, between 1999 and 2004, we assembled a representative sample of community dwellers of Porto, an urban centre in the northwest of Portugal, with approximately 300,000 inhabitants at the time. We selected Households by random digit dialling of landline telephones. Within each household, selected by simple random sampling a permanent resident aged 18 years or more and not replaced refusals. The proportion of participation was 70%, and the final sample size was 2485 individuals. Of the 633 participants with age between 65 and 85 years old, 586 completed the assessment at baseline. The follow-up evaluation took place in two waves, part of the participants (*N* = 221) were evaluated during the first follow-up, between 2005 and 2008, and the others were evaluated only on the second follow-up (*N* = 66), between 2013 and 2015 (Fig. 1).

### Data collection and definition of variables

Trained interviewers performed a face-to-face questionnaire which collected data on sociodemographic and behavioural characteristics [29] and administered the Mini-Mental State Exam (MMSE) at the beginning of each interview [30]. Education was recorded as completed years of schooling and further categorized into three groups: zero years, one to 9 years and more than 10 years of schooling. Civil status was categorized in two groups: married or living together, and not-married (divorced, single or widowed). Professional status was considered to be either working (participants employed), retired (considering retirement as a direct transition between a situation of full employment and a situation where the individual is entirely inactive and where most of his resources consist of pension benefits), or housewives. There were no unemployed participants. Cognitive impairment was evaluated using the MMSE [30], with cut-off points adjusted by years of schooling validated for the Portuguese population: 22 for 0–2 years; 24 for 3–6 years and 27 for seven or more years of schooling [31]. Subjects with an MMSE score below the age and education adjusted cut-off point were considered to have cognitive impairment. The MMSE is the most cited small-sized scale used for dementia and cognitive impairment assessment and is thought to be a reliable and valid test for cognitive impairment [30, 32].

### Prevalence evaluation

At the baseline evaluation, 633 participants were aged 65 to 85 years old, but we excluded 47 subjects due to missing information on the Mini-Mental State Exam (MMSE). The final sub-sample was 586 participants (Table 1) with 71.95 years (± 4.84 SD) mean age; 355 were women (60.6%); 57 (9.7%) had no education, 432 (73.7%) had one to 9 years of education, and 97 (16.6%) had more than 10 years (16.6%) of schooling; 350 (59.7%) were married or living in civil union; 464 (79.2) were retired.

**Table 1:** Socio-demographic characteristics of participants

Characteristic	Baseline	Follow-up	Lost to follow-up	*P* value
N	586	287	208	
**Sex**				
Female	355 (60.6)	169 (58.9)	119 (57.2)	0.709
Male	231 (39.4)	118 (41.1)	89 (42.8)	
**Age (years)**				
[65–69]	216 (36.9)	125 (43.6)	67 (32.2)	0.010*
[70–74]	200 (34.1)	100 (34.8)	70 (33.7)	
[75–79]	120 (20.5)	46 (16.0)	50 (24.0)	
[80–85]	50 (8.5)	16 (5.6)	21 (10.1)	
**Education**				
0	57 (9.7)	17 (5.9)	14 (6.7)	0.751
[1–9]	432 (73.7)	219 (76.3)	162 (77.9)	
≥ 10	97 (16.6)	51 (17.8)	32 (15.4)	
**Marital Status**				
Married/Civil Union	350 (59.7)	180 (62.7)	118 (56.7)	0.179
Single, divorced, widower	236 (40.3)	107 (37.3)	90 (43.3)	
**Employment Status**				
Work	50 (8.5)	26 (9.1)	18 (8.7)	0.626
Retired	464 (79.2)	233 (81.2)	164 (78.8)	
Housewives	71 (12.1)	28 (9.8)	26 (12.5)	

Legend: Data are *n* (%); *P*-value compares follow-up to lost to follow up, obtained with Chi-square test

*Significant at *p* < 0.05

### Incidence evaluation

There were two follow-up evaluations where the participants completed a questionnaire and had a physical examination. The first follow-up was between 2005 and 2008 and the second follow-up between 2013 and 2015. Some participants were evaluated only during the 1st or the 2nd follow-up. Of the initial 586 eligible participants, there were overall 208 losses with 103 (49.5%) deaths, 51 (24.5%) refusals, 26 (12.5%) were not possible to contact and 28 (13.5%) missed without justification. We re-evaluated a total of 287 participants (mean follow-up of 6.2 years, SD 4.30 years). There were no significant differences regarding sex, education, civil status or employment status between follow up participants and those lost to follow-up (Table 1). However, participants lost to follow-up were older (72.58 vs 71.09 mean age in years).

### Competing risk model

During the follow-up, in the disease/death process, often more than one type of event plays a role. We are interested as the first event a diagnosis of cognitive impairment. However, death may prevent the event of interest from occurring, because the person died before the diagnosis of cognitive impairment. Therefore, death is a competing risk of cognitive impairment and may substantially change the risk of disease diagnosis. Death substantially reduces the probability of being diagnosed with cognitive impairment, and hence is treated as a competing risk event when calculating cognitive impairment incidence [33, 34]. Ignoring death as a competing risk or treat it as no informative censored observations will lead to a bias in the standard methods for estimate the probability of the event [35] such as the Kaplan-Meier estimate [36]. The assumption of independence of the time to event and the censoring distributions is violated and then violates one of the fundamental assumptions of the Kaplan-Meier estimator. We considered the time of event as the time from entering in the cohort to the first event, cognitive impairment or death, during the follow-up.

The cumulative incidence function (CIF) allows for estimation of the incidence of the occurrence of an event while taking competing risk into account [37, 38]. This allows one to estimate incidence in a population where all competing events must be accounted for in clinical decision making. It denotes the probability of experiencing the *kth* event before time t and before the occurrence of a different type of event, i.e., for instance, the probability of experience death before 70 years old, before the occurrence of the cognitive impairment. The CIF has the desirable property that the sum of the CIF estimates of the incidence of each of the individual outcomes will equal the CIF estimates of the incidence of the composite outcome consisting of all of the competing events [39].

We performed the competing risks analysis to the 495 participants at risk of incidence of cognitive impairment, excluding those 105 that refused to participate, missed or were impossible to contact along all period of the follow-up. From these 390, 103 died before cognitive impairment diagnosis, 48 were diagnosed with cognitive impairment and 239 were still alive without cognitive impairment diagnosis.

### Statistical analysis

We assessed differences in the prepositions using the Chi-Square test. Losses to follow-up were compared to participants in the follow-up using the Chi-Square test. Calculated crude incident rates dividing the number of events by total number of person-years at risk. Counted time at risk as the time in years between the baseline evaluation and the last follow-up that each participant attended and taking into consideration the full length of time for subjects who remained free of cognitive impairment, and estimate time of onset of cognitive impairment being set to the midpoint between the baseline and follow-up observation waves for those participants who did develop the disease. Poisson generalized linear models were used to determine confidence intervals, with Log of time at risk as to the offset. We tested the possible interaction of each explanatory variable with age, sex, education and retirement status was tested. Sex-, age-, education- and education- adjusted OR and RR were estimated. Standardized prevalent and incident rates were calculated for the Portuguese population using data from the last census, in 2011 [40], and for European population using data from the standard European population, 2013 [41]. The CIFs were estimated in R using the cuminc function in the *cmprsk* R [42] package which uses the cumulative incidence function introduced by Kalbfleisch and Prentice [38].We used the Gray’s test [43] for equality of CIFs across groups. We assessed differences in the MMSE mean score reduction of participants with and without cognitive impairment with T-test for independent samples. The remaining statistical analyses were performed using SPSS**®** version 21. We include the Box Plot of the Mini-Mental State Examination score of the population at baseline evaluation and of the participants with or without cognitive impairment at the follow-up evaluation as Supplementary material (Figure S1).

## Results

### Prevalence evaluation

The crude prevalence of cognitive impairment was 15.5% (95% CI: 12.7–18.7) at baseline. The standardized prevalence rate for the Portuguese population was 16.9% and for the standard European population was 16.5%. Prevalence was lower in men (10.4%; 95% CI: 6.7–15.1) than in women (18.9; 95% CI: 14.9–23.3), with the odds of presenting cognitive impairment, adjusted for age and education, of 0.95 (95% CI: 0.89–1.01).

The prevalence of cognitive impairment increases with age, being higher at 80–85 years than 65–69 years (26.0; 95% CI: 17.3–40.2 vs 11.1; 95% CI: 9.3–17.2), with the odds for cognitive impairment, adjusted for sex and education, being 1.14 higher.

The cognitive impairment prevalence is higher for participants with zero years of schooling (45.6; 95% CI: 32.4–59.3) and slightly higher for participants with more than 10 years than for participants with one to 9 years (14.4; 95% CI 8.1–23.0 vs 11.8; 95% CI 8.9–15.2) with statistically significant differences.

Not-married participants had a higher prevalence of cognitive impairment (16.5; 95% CI: 12.0–21.9 vs 14.9; 95% CI: 11.3–19.0), but the difference was not statistically significant after adjustment for age and education.

Retired participants had a higher prevalence of cognitive impairment than the working participants (14.4; 95% CI: 11.4–18.0 vs 12.0; 95% CI: 4.6–24.3) and housewives have the highest prevalence (23.9; 95% CI: 14.6–35.5) but without statistically significant differences. (Table 2).

**Table 2:** Observed prevalence of cognitive impairment by socio-demographic characteristics

Characteristics	n	Prev. % (95% CI)	*p*-value	OR (95% CI) Adjusted
**Sex**				
Female	67	18.9 (14.9–23.3)	0.006*	1 [reference]^a^
Male	24	10.4 (6.7–15.1)		0.95 (0.89–1.01)
**Age (years)**				
[65–69]	24	11.1 (9.3–17.2)	0.026*	1 [reference]^b^
[70–74]	30	15.0 (11.0–21.1)		1.03 (0.97–1.10)
[75–79]	24	20.0 (13.3–28.3)		1.10 (1.02–1.19)
[80–85]	13	26.0 (17.3–40.2)		1.14 (1.03–1.27)
**Education**				
0	26	45.6 (32.4–59.3)	0.000*	1 [reference]^c^
[1–9]	51	11.8 (8.9–15.2)		0.72 (0.66–0.80)
≥ 10	14	14.4 (8.1–23.0)		0.74 (0.67–0.84)
**Marital Status**				
Married/Civil Union	52	14.9 (11.3–19.0)	0.584	1 [reference]^d^
Single, divorced, widower	39	16.5 (12.0–21.9)		0.96 (0.90–1.02)
**Employment Status**				
Work	6	12.0 (4.6–24.3)	0.471	1 [reference]^a^
Retired	67	14.4 (11.4–18.0)		99.6 (0.90–1.10)
Housewives	17	23.9 (14.6–35.5)		1.07 (0.94–1.22)

Legend: *Prev* Prevalence, *OR* odds ratio. 95% CI: 95% confidence interval

^a^adjusted for age and education

^b^adjusted for sex and education

^c^adjusted for sex and age

^d^adjusted for sex, age and education

* Significant at *p* < 0.05

### Incidence evaluation

During the study protocol, 48 individuals developed cognitive impairment, an incidence rate of 26.97 per 1000 person-years (95% CI: 20.3–35.8). The standardized incidence rate using the Portuguese population was 35.7 per 1000 person-years and using the standard European population was 34.4 per 1000 person-years.

The incidence of cognitive impairment was higher in women (33.8 per 1000 person-years; 95% CI: 24.2–47.4) than men (18.0 per 1000 person-years; 95% CI: 10.7–30.5) and increasing with age at 80–85 years old (66.0 per 1000 person-years; 95% CI: 27.5–158.7) vs 65–69 years old (21.1 per 1000 person-years; 95% CI: 13.5–33.1).

As observed on baseline prevalence, the incidence is higher for participants with zero years of schooling (126.4; 95% CI: 68.0–234.8) and almost the same for participants with 1 to 9 years and more than 10 years (21.6; 95% CI: 15.0–31.0 vs 25.3; 95% CI 13.2–48.7).

Not married participants have a higher incidence rate of cognitive impairment (32.5; 95% CI: 21.4–49.4 vs 23.6; 95% CI: 16.1–34.6), but the difference did not reveal statistically significant differences after adjusting for age and education.

Retired participants have a higher incidence of cognitive impairment than working participants (30.0; 95% CI: 21.3–37.9 vs 18.1; 95% CI: 6.8–48.3) but without statistically significant differences. (Table 3).

**Table 3:** Observed incidence of cognitive impairment by socio-demographic characteristics

Characteristics	n	Incidence (95% CI) per 1000 person-years	*p*-value	RR (95% CI) Adjusted
**Sex**				
Female	34	33.8 (24.2–47.4)	0.084	1 [reference]^a^
Male	14	18.0 (10.7–30.5)		0.66 (0.35–1.27)
**Age (years)**				
[65–69]	19	21.1 (13.5–33.1)	0.142	1 [reference]^b^
[70–74]	16	25.8 (15.8–42.2)		1.29 (0.66–2.52)
[75–79]	8	43.5 (21.7–86.9)		2.20 (0.96–5.05)
[80–85]	5	66.0 (27.5–158.7)		2.01 (0.72–5.58)
**Education**				
0	10	126.4 (68.0–234.8)	0.005*	1 [reference]^c^
[1–9]	29	21.6 (15.0–31.0)		0.21 (0.10–0.47)
≥ 10	9	25.3 (13.2–48.7)		0.25 (0.10–0.65)
**Marital Status**				
Married/Civil Union	26	23.6 (16.1–34.6)	0.296	1 [reference]^d^
Single, divorced, widower	22	32.5 (21.4–49.4)		1.03 (0.55–1.93)
**Employment Status**				
Work	4	18.1 (6.8–48.3)	0.911	1 [reference]^a^
Retired	40	30.0 (21.3–37.9)		1.30 (0.44–3.79)
Housewives	4	22.3 (24.2–47.4)		0.79 (0.19–3.31)

Legend: *RR* relative risk. 95%, *CI* 95% confidence interval

^a^adjusted for age and education

^b^adjusted for education and sex

^c^adjusted for age and sex

^d^adjusted for sex, age and education

*Significant at *p* < 0.05

**Fig. 2:**
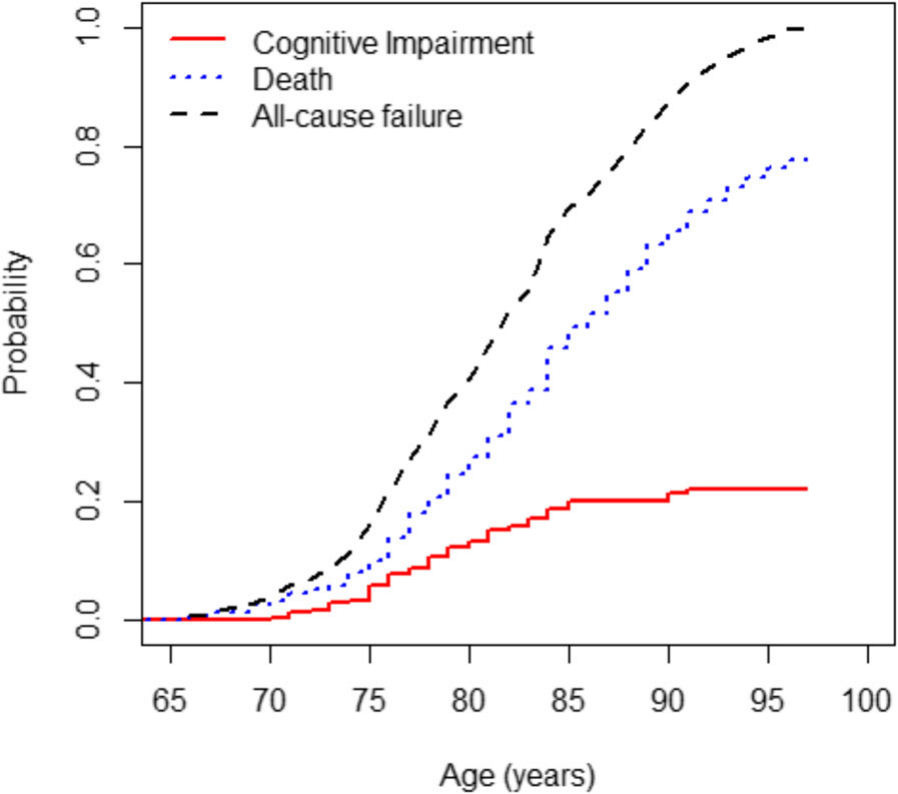
Cumulative incidence functions. The solid line shows the cumulative incidence of cognitive impairment. The dotted line shows the competing risk event, i.e. death occurring prior to the cognitive impairment. The dashed line shows the cumulative incidence function of all-cause failure, i.e. the sum of the cumulative incidences of the 2 cause-specific failures

**Fig. 3:**
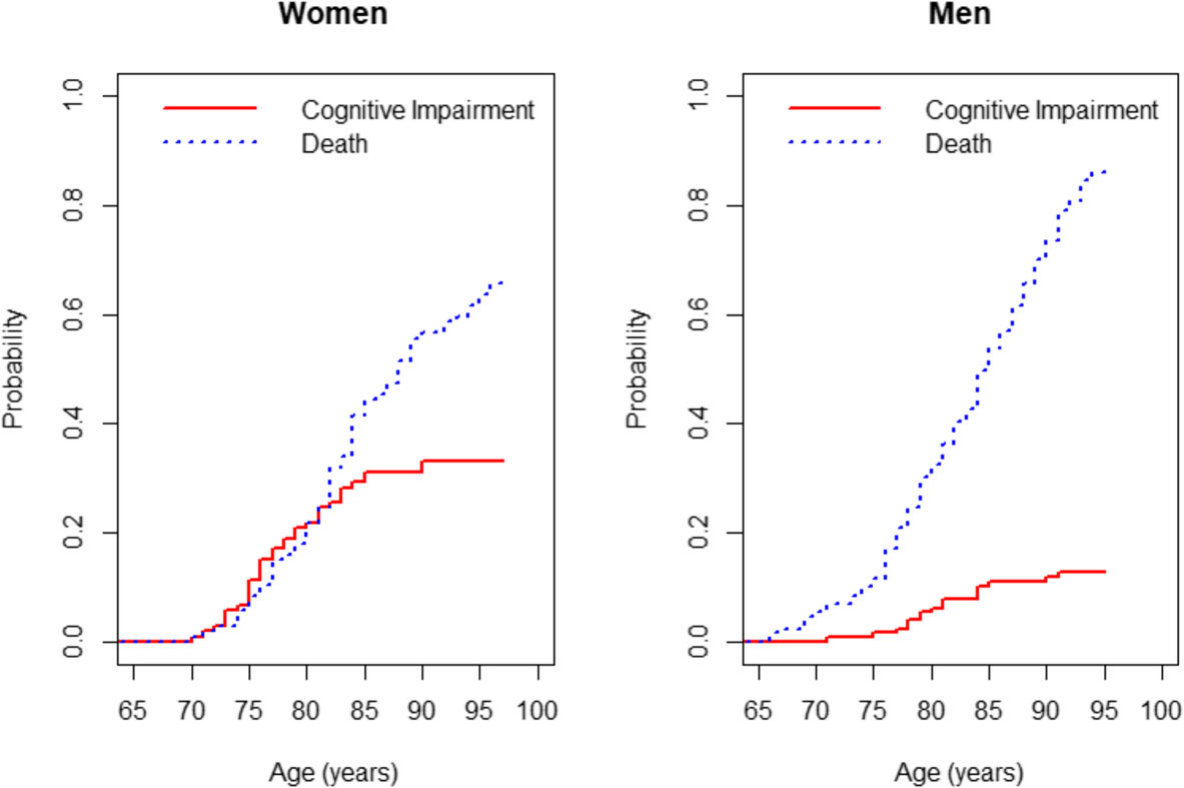
Cumulative incidence functions by sex. Left panel: The solid line shows the cumulative incidence of cognitive impairment, for women. Right panel: The dotted line shows the competing risk event, i.e. death occurring prior to the cognitive impairment, for Men

The crude cumulative incidences of cognitive impairment and death in the overall sample are described in Fig. 2, along with the incidence of the composite outcome of all-cause of failure (death or cognitive impairment). The cumulative incidence of all-cause failure is equal to the sum of the cumulative incidences of the 2 cause-specific failures. Although the cumulative incidence of death before the cognitive impairment exceeded that of cognitive impairment diagnosis at each point in time, the incidence of cognitive impairment was not negligible in this population. In the group analysis by sex, the cumulative incidence curves for women and men were statistically different for cognitive impairment before death (*P*-value 0.0008), and for death before cognitive impairment (*P*-value 0.0004). The estimated CIFs for each cause of failure by sex displayed in Fig. 3 presented notable differences. In women, from 73 years old to 80 years old, the cumulative incidence of cognitive impairment is higher that its competitive event, while for the men, the incidence of death before cognitive impairment is higher in all points in time when compared to the cognitive impairment diagnosis, following the same trend as when analysing the whole sample. The estimates of death before cognitive impairment diagnosis of the women exceeds the estimates of cognitive impairment diagnosis in the point’s time after 80 years old. The slope of the curves are quite similar in women and men until 70 years − 75 years old, however, in the men it can be observed a higher probability of death before cognitive impairment than in the women.

Participants with cognitive impairment had an average MMSE score reduction of 5.33 (SD 3.64), while participants without cognitive impairment had an average of 0.44 reduction (SD = 1.84) (*p* = 0.000) over the time at risk of 6.2 mean years (± 4.30 years).

## Discussion

In this urban population with 65 to 85 years, the prevalence of cognitive impairment was 15.5%. It was higher in women, in not-married participants, and retired participants, it increased with age and decreased with education years.

Previous studies with Portuguese population samples reported a prevalence of cognitive impairment between 9.3 and 12.0% [10, 25, 26] using younger participants, over 50 years old on the first study and over 55 years old on the other two. Both the studies which used the MMSE as a screening test, Nunes et al. [25] and Ruano et al. [26] complemented the results with a neurologist evaluation. It is worth to point out that Ruano et al. [26] report the 2015 prevalence for the EpiPorto cohort with the present study focusing on the period from 1999 to 2004 and that only in the current study was the incidence of cognitive impairment in this population ascertained (1999–2015).

The prevalence estimate found in our study is within the estimated interval for the European population using ascertainment approaches similar to ours [9, 20].

As previously reported cognitive impairment prevalence was higher in women than in men [9, 10, 44], with studies pointing out hormonal causes, namely the loss of estrogens in women, to justify this difference [45]. It increased in frequency with increasing age between the ages of 65 and 85 years, which is in accordance to other studies that associated ageing with cognitive decline and dementia [9, 10, 44, 46] and it is associated with low levels of education, possibly due to the higher cognitive reserve of the ones with more years of education [9, 44, 47]. Participants without schooling have a higher prevalence of cognitive impairment than participants with at least some schooling. Participants with more than 10 years of schooling have a higher prevalence of cognitive impairment compared with those between one and 9 years of schooling. However, this may be attributable to their higher mean age and presence of more participants with 80–85 years old (13.4% vs 6.9%). When we determined the odds ratio, adjusted for sex and age, the risk is almost the same (0.72 vs 0.74) between both groups and statistically different for the group without any schooling. It was also slightly higher for not-married participants [9, 44, 48] despite not reaching statistical significance and we did not find differences between retired and non-retired participants (Table 2).

For 6.2 mean years of follow-up time, we observed that the incidence rate of cognitive impairment was 26.97 per 1000 person-years. The standardized incident rate for the Portuguese population was 35.7 and for the standard European population was 34.4 per 1000 person-years. The cognitive impairment incidence we found in our sample is lower than estimates for other European countries [20, 21, 49] and North America, [22, 23] however this might be due to the older populations and different cognitive impairment definitions used in these studies.

In concordance with other studies [22, 24, 49, 50], we also observed a trend towards increased incidence with older age, and higher in women albeit without reaching statistical significance. These findings were also achieved when considering death as a competing risk of cognitive impairment.

Participants with zero years of schooling have a higher incidence of cognitive impairment than the ones with schooling, which is in concordance to the impact of education years reported in previous findings [20, 51].

Non-married participants have a non-significant trend towards a higher incidence of cognitive impairment, which could be explained by the memory and cognition stimulation of the married participants [52].. For employment status, we observe that retired participants have a trend towards a higher incidence of cognitive impairment than the ones which are still working, after adjusting for age and education, which may indicate a protective effect of working, as described before [8, 53]. It would be interesting to conduct further work to try to determine if indeed working has a protective effect on cognitive decline or if other social dimensions involved in being employed mediates this effect.

The average MMSE score reduction is higher in participants with cognitive impairment compared with other participants, which demonstrates a more pronounced cognitive loss on the first ones that must be taken into account when defining preventive measures in the Health System.

A previous study [26] has found that one of the major causes of cognitive impairment in this population stems from vascular disease, as such we suggest that help managing blood pressure and an increase in physical activity, if targeted to these groups, could lead to significant public health improvements.

In Portugal, in the period under analysis, from 1999 to 2015, the demographic characteristics of the population over 65 years old changed and, according to PORDATA, the percentage of older people increased from 15.9 to 20.5% [54], and the number of people without education decreased by 36.7% and those with higher education, increased by 247.7% [55]. The increased education may have contributed to the decrease in the prevalence of cognitive impairment from 15.5 to 9.3% [26], despite some methodological differences in the two studies in the EpiPorto cohort discussed above. The increase in schooling will mitigate the effect of increasing average life expectancy, but it should be taken other measures because by 2050, Portugal will be one of the European countries with a higher percentage of older people and with the highest old-age dependency ratio.

The main strengths of our study are the population-based cohort and the long-term prospective study design as well as the use of MMSE published cut-off points adjusted for education. This study provides an estimate of the prevalence and incidence of cognitive impairment in an elder western European cohort providing essential data to target public health strategies accurately.

Some methodological limitations may have overestimated the results, namely the inability to diagnose dementia, meaning that we could not exclude participants with dementia from the study, which may have overestimated the prevalence of cognitive impairment at baseline. Mortality as a competing risk may have overestimated the incidence of cognitive impairment. Participants lost-to-follow-up where older and with lower mean MMSE scores than the participants and this could have underestimated the incidence calculations.

## Conclusion

This study reports a prevalence of cognitive impairment of 15.5% and an incidence of 26.97 per 1000 person-years in a cohort of city dwellers from Porto, Portugal, aged 65 to 85 years old. Women and elders without schooling have a higher risk of developing cognitive impairment, and this risk increases with ageing. This study highlights the need to develop preventive health measures targeted to these groups to help maintain brain health with ageing. In our study we found that neither retirement nor marital status have a significant effect on cognitive impairment.

## Supplementary Information

**Additional file 1:** **Figure S1.** Box Plot of the Mini-Mental State Examination score of the population at baseline evaluation and participants with or without cognitive impairment at the follow-up evaluation.

## Data Availability

The datasets used and analysed during the current study are available from the corresponding author on reasonable request.

## Abbreviations

MMSE: Mini-Mental State Examination
CIF: Cumulative incidence function
OR: Odds ratio
RR: Relative Risk
