# Supplementary figures and images for: Prevalence and incidence of cognitive impairment in an elder Portuguese population (65–85 years old)
Source: BMC Geriatr. 2020 Nov 16;20:470. doi: 10.1186/s12877-020-01863-7 (PMC7667782; doi:10.1186/s12877-020-01863-7)

MMSE Score

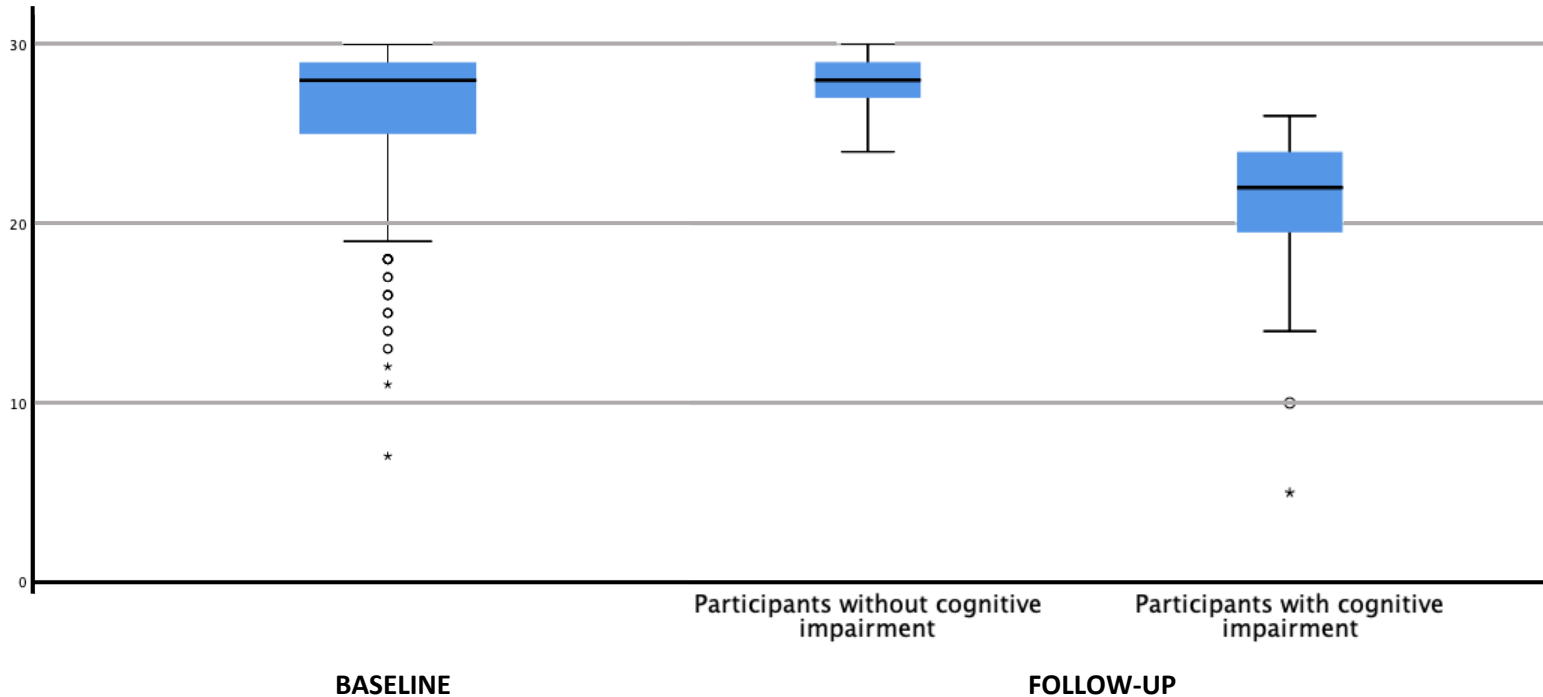

Supplement: Supplementary file 1 — Additional file 1: Figure S1. Box Plot of the Mini-Mental State Examination score of the population at baseline evaluation and participants with or without cognitive impairment at the follow-up evaluation. [file 12877_2020_1863_MOESM1_ESM.pdf]
